# Association of triglyceride glucose index with the risk of acute kidney injury in patients with coronary revascularization: a cohort study

**DOI:** 10.1186/s13098-024-01358-0

**Published:** 2024-05-28

**Authors:** Yue Shi, Hangyu Duan, Jing Liu, Xiujie Shi, Mingming Zhao, Yu Zhang

**Affiliations:** 1 Department of Nephrology, Xiyuan Hospital, China Academy of Chinese Medical Sciences, Beijing, 100091 China; 2 Beijing University of Chinese Medicine, Beijing, 100029 China

**Keywords:** Triglyceride glucose index, TyG, Coronary revascularization, Acute kidney injury, MIMIC-IV, Cohort study

## Abstract

**Background:**

The triglyceride glucose (TyG) index is a novel and reliable alternative marker for insulin resistance. Previous studies have shown that TyG index is closely associated with cardiovascular outcomes in cardiovascular diseases and coronary revascularization. However, the relationship between TyG index and renal outcomes of coronary revascularization is unclear. The purpose of this study was to investigate the correlation between TyG index and the risk of acute kidney injury (AKI) in patients with coronary revascularization.

**Methods:**

A retrospective cohort study was conducted to select eligible patients with coronary revascularization admitted to ICU in the medical information mart for intensive care IV (MIMIC-IV). According to the TyG index quartile, these patients were divided into four groups (Q1-Q4). The primary endpoint was the incidence of AKI, and secondary endpoints included 28-day mortality and the rate of renal replacement therapy (RRT) use in the AKI population. Multivariate Cox regression analysis and restricted cubic splines (RCS) were used to analyze TyG index association with AKI risk. Kaplan-Meier survival analysis was performed to assess the incidence of endpoints in the four groups.

**Results:**

In this study, 790 patients who underwent coronary revascularization surgery were included, and the incidence of AKI was 30.13%. Kaplan-Meier analysis showed that patients with a high TyG index had a significantly increased incidence of AKI (Log-rank *P* = 0.0045). Multivariate Cox regression analysis showed that whether TyG index was a continuous variable (HR 1.42, 95% CI 1.06–1.92, *P* = 0.018) or a categorical variable (Q4: HR 1.89, 95% CI 1.12–3.17, *P* = 0.017), and there was an independent association between TyG index and AKI in patients with coronary revascularization. The RCS curve showed a linear relationship between higher TyG index and AKI in this particular population (*P* = 0.078). In addition, Kaplan-Meier analysis showed a significantly increased risk of RRT application in a subset of AKI patients based on quartiles of TyG index (*P* = 0.029).

**Conclusion:**

TyG index was significantly associated with increased risk of AKI and adverse renal outcomes in patients with coronary revascularization. This finding suggests that the TyG index may be useful in identifying people at high risk for AKI and poor renal outcomes in patients with coronary revascularization.

## Introduction

Coronary artery disease (CAD) is a multifactorial fatal disease that has affected 244.11 million people worldwide and is an increasing global public health burden [1–3]. Although superior evidence-based strategies such as optimizing medical treatment have been widely developed and applied in recent years, coronary revascularization surgery is still recommended in patients with coronary heart disease with severe coronary stenosis or obstruction and poor response to medical treatment. Currently, percutaneous coronary intervention (PCI) and coronary artery bypass grafting (CABG) are the most common strategies for coronary revascularization [4]. However, PCI and CABG are associated with an increased risk of acute kidney injury (AKI), with the incidence of AKI reported to be approximately 3–20% after PCI and up to 36% after CABG [5–9]. AKI is a common complication of CABG and PCI, and its development predicts the risk of long-term adverse health consequences, such as length of hospital stay, death, heart failure, and progression of kidney disease [10, 11]. Therefore, it is necessary to further explore the appropriate risk stratification for AKI in patients who have undergone coronary revascularization procedures and personalize their treatment.

Insulin resistance (IR) is a decrease in the efficiency of insulin to promote glucose uptake and utilization and plays an important role in metabolic signaling pathways in multiple organs such as the heart and kidney [12, 13]. As the gold standard technique for measuring IR, the hyperinsulinemia - normal blood glucose clamp has limited clinical application due to its complex operation. Recently, the triglyceride glucose (TyG) index has been recognized as a novel and reliable surrogate marker for IR. TyG index, derived from fasting blood glucose (FBG) and fasting triglycerides (TG), was calculated by Ln (fasting TG [mg/dL] × FBG [mg/dL]/2). Previous studies have shown that TyG index is significantly associated with the incidence of CAD, including coronary artery stenosis or calcification, and carotid atherosclerosis [14, 15]. Meanwhile, there was a linear relationship between TyG index and CAD severity [16]. Furthermore, the TyG index showed promising prognostic potential in patients with acute coronary syndrome who underwent PCI and/or CABG, patients with type 2 diabetes (T2DM) who underwent PCI, and patients with heart failure [4, 17, 18]. However, most scholars usually focus on the predictive potential of TyG index for cardiovascular outcome events, but ignore the association between TyG index and the risk of AKI in patients after coronary revascularization. Therefore, we aimed to evaluate the effect of TyG index on risk stratification of AKI in patients with coronary revascularization, thereby providing new clues for early prediction of AKI.

## Methods

### Data sources

The data were obtained from the medical information mart for intensive care IV (MIMIC-IV) database (https://mimic.mit.edu). This database records data on adults hospitalized in the ICU at Beth Israel Deaconess Medical Center between 2008 and 2019. Data for this study were obtained from publicly available sources with de-identified information, thus exempting ethical approval and informed consent requirements. The first author Y.S. completed an online course at the National Institutes of Health and passed the Human Research Participant Protection Examination, obtaining permission for a collaborative institutional training program and the use of the MIMIC-IV database.

### Study population

Adult patients undergoing coronary revascularization procedures, including PCI and CABG, who were admitted to the ICU for the first time were included. AKI is defined by Kidney Disease: Improving Global Outcomes guideline criteria [19]. AKI was defined as an increase in serum creatinine (Scr) level of ≥ 0.3 mg/dl within 48 h or ≥ 1.5 times the baseline within 7 days. Exclude patients who lack TG and FBG data within 24 h of ICU admission.

### Data collection

Demographic information, vital signs, sequential organ failure assessment (SOFA) scores, laboratory measures, comorbidities, interventions were extracted using structured query language (SQL) running PostgresSQL (version 13.7.2). Demographic information included sex age, body mass index (BMI). Vital signs included heart rate and blood pressure, respiratory rate. Laboratory parameters were selected as initial values within 24 h after the first ICU admission, including white blood cell (WBC) count, red blood cell (RBC) count, platelet count, hemoglobin, red blood cell distribution width (RDW), Scr, blood urea nitrogen (BUN), albumin, fasting blood glucose (FBG), serum sodium, serum potassium, TG, total cholesterol (TC), low-density lipoprotein (LDL), and high-density lipoprotein (HDL). Comorbidities included heart failure, chronic kidney disease (CKD), hypertension, diabetes mellitus (DM), sepsis, malignancy. Interventions included renal replacement therapy (RRT), diuretics, and vasoactive drugs, which were derived from data recorded within 24 h of ICU admission.

### Management of abnormal and missing values

Variables with outliers were processed by the winsorize method using the STATA winsor2 command with 1% and 99% as cutoff points. For missing values, we employed a multiple imputation approach. Variables with more than 25% missing values were excluded, such as lactate and C-reactive protein.

### Study grouping and outcome endpoints

The TyG index was calculated using the following formula: Ln (fasting TG [mg/dL]× FBG [mg/dL]/2). All patients were divided into four groups according to TyG quartile. Namely, group Q1 (5.17 ≤ TyG ≤ 8.69), group Q2 (8.69 < TyG ≤ 9.15), group Q13 (9.15 < TyG ≤ 9.58) and group Q4 (9.58 < TyG ≤ 11.44). The primary endpoint was the incidence of AKI. Secondary endpoints were 28-day mortality in the overall study population and AKI population and RRT application in AKI population.

### Statistical analysis

Normally distributed continuous variables were presented as mean ± standard deviation and compared using one-way ANOVA or t-test. Continuous variables with skewed distributions were presented as medians (quartile) and compared by Kruskal-Wallis H or Mann-Whitney U test. Categorical variables were presented as frequencies and percentages and compared using the Chi-square test or Fisher ‘s exact test.

Cox proportional hazards models were used to calculate hazard ratio (HR) and 95% confidence interval (CI) for TyG index and AKI, adjusted for multiple confounding variables. Confounding variables were selected by stepwise regression (*P* < 0.05 for selection) (Model 1: unadjusted; Model 2: adjusted for sex, age, and BMI; Model 3: On the basis of model 2, albumin, Scr, BUN, WBC, RBC, platelet, hemoglobin, RDW, LDL, HDL, TG, vasoactive drugs, diuretics, vasoactive drugs, diuretics, hypertension, heart failure, DM, CKD, and RRT were added). To prevent multicollinearity, we excluded variables with variance inflation factors greater than 5 from the model. Kaplan-Meier survival analysis was used to assess the incidence of AKI, the application rate of RRT, and 28-day mortality between groups based on TyG index, and Log-Rank test was used to determine differences among the four groups. The potential dose-response association between TyG index and AKI incidence was investigated using adjusted restricted cubic splines (RCS). In addition, subgroup analysis and interaction analysis were performed according to age, sex, BMI, CKD, heart failure, hypertension and DM.

Data analysis was performed using R software (Version 4.2.0) and STATA software (Version 16.0). The two-tailed tests indicated that *P* < 0.05 was considered statistically significant.

## Results

### Baseline characteristics

**Fig. 1:**
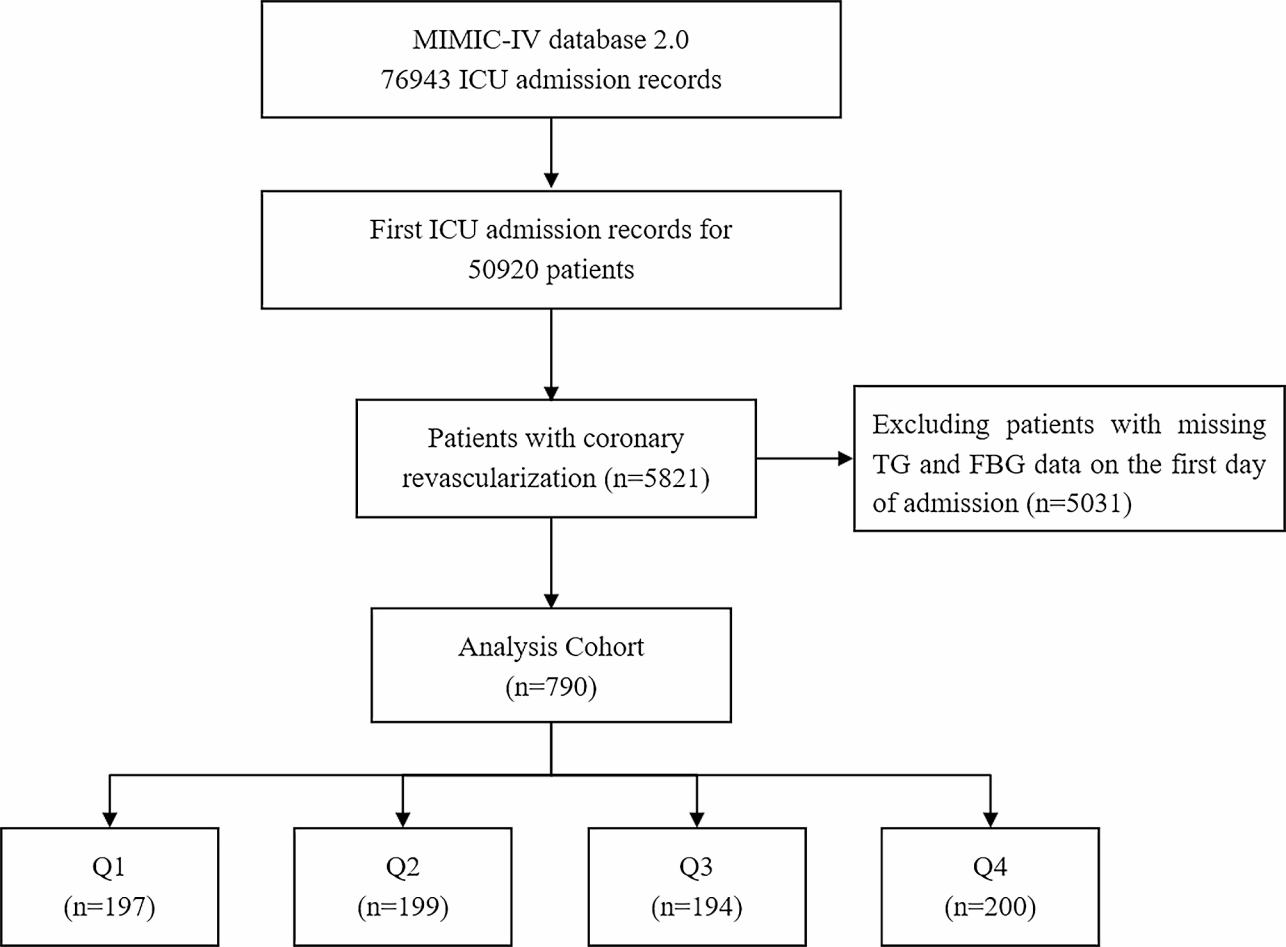
Flow chart of patient selection

According to various inclusion and exclusion criteria, a total of 790 patients undergoing coronary revascularization procedures were included in this study, and the patient screening flow chart was shown in Fig. 1. These patients were divided into four groups according to TyG index quartiles and their baseline characteristics were shown in Table 1. Patients with a higher TyG index were generally younger, had higher BMI, SOFA scores, faster heart rates, a higher prevalence of DM, and a higher proportion of RRT and vasoactive drug use. In terms of laboratory parameters, WBC, Scr, BUN, FBG, potassium, TG, and TC were higher in the higher TyG index group than in the lower TyG index group, while sodium and HDL were the opposite. The incidence of AKI (25.38% vs. 26.13% vs. 29.38% vs. 39.50%, *P* = 0.007) increased with increasing TyG index.

**Table 1:** Baseline characteristics according to TyG quartiles

Variables	Overall	Q1 (5.17–8.69)	Q2 (8.69–9.15)	Q3 (9.15–9.58)	Q4 (9.58–11.44)	*P*-value
Participants	790	197	199	194	200	
Age (years)	67.65 (58.59, 76.99)	69.56 (61.39, 80.84)	68.50 (58.25, 78.22)	66.21 (58.30, 75.72)	66.03 (57.08, 73.34)	0.002
Male, *n* (%)	567 (71.77)	136 (69.04)	141 (70.85)	153 (78.87)	137 (68.50)	0.083
BMI (kg/m^2^)	29.02 (25.72, 32.26)	27.62 (24.93, 31.24)	28.52 (35.47, 31.25)	29.34 (26.12, 32.58)	30.46 (26.80, 33.66)	<0.001
Vital signs						
HR (beats/min)	79.68 (71.45, 87.04)	77.36 (70.15, 83.82)	78.79 (69.87, 86.67)	79.82 (71.85, 85.81)	82.56 (74.81, 89.42)	<0.001
SBP (mmHg)	112.68 (105.68, 120.83)	112.76 (106.04, 126.61)	113.11 (103.36, 120.72)	112.49 (106.24, 120.90)	111.97 (105.32, 121.37)	0.852
DBP (mmHg)	61.35 (55.00, 69.94)	60.67 (55.53, 69.00)	62.21 (55.62, 71.01)	61.38 (54.59, 70.00)	60.65 (54.60, 69.57)	0.615
RR (beats/min)	18.08 (16.38, 20.11)	18.02 (16.31, 20.04)	18.00 (16.23, 20.02)	17.84 (16.18, 19.56)	18.52 (16.79,20.85)	0.103
SOFA	3.00 (1.00, 6.00)	3.00 (1.00, 5.00)	2.00 (1.00, 5.00)	3.00 (1.00, 7.00)	4.00 (2.00, 7.00)	0.001
Comorbidities, *n* (%)						
Heart failure	257 (32.53)	59 (29.95)	61 (30.65)	63 (32.47)	74 (37.00)	0.434
CKD	119 (15.06)	33 (16.75)	29 (14.57)	22 (11.34)	35 (17.50)	0.320
Hypertension	298 (37.72)	66 (33.50)	76 (38.19)	79 (40.72)	77 (38.50)	0.511
DM	125 (15.82)	17 (8.63)	22 (11.06)	30 (15.46)	56 (28.00)	0.001
Sepsis	287 (36.33)	63 (31.98)	56 (28.14)	75 (38.66)	93 (46.50)	0.320
Malignancy	105 (13.29)	32 (16.24)	28 (14.07)	23 (11.86)	22 (11.00)	0.415
Interventions, *n* (%)						
RRT	40 (5.06)	7 (3.55)	7 (3.52)	5 (2.58)	21 (10.50)	0.001
Diuretics	460 (58.23)	105 (53.30)	113 (56.78)	117 (60.31)	125 (62.50)	0.263
Vasoactive drugs	169 (21.39)	46 (23.35)	26 (13.07)	40 (20.62)	57 (28.50)	0.002
Laboratory tests						
RBC (m/uL)	3.76 (3.10, 4.35)	3.75 (3.20, 4.34)	3.91 (3.09, 4.40)	3.68 (3.02, 4.32)	3.78 (2.99, 4.41)	0.726
WBC (K/uL)	11.50 (8.80, 15.00)	10.50 (8.00, 13.60)	11.50 (8.90, 14.60)	12.10 (9.10,15.40)	12.50 (9.40, 16.85)	<0.001
Hemoglobin (g/dL)	11.80 (9.70, 13.50)	11.90 (10.10, 13.20)	12.20 (9.70, 13.50)	11.60 (9.60, 13.70)	11.30 (9.40, 13.40)	0.661
RDW (%)	13.50 (12.90, 14.30)	13.40 (13.00, 14.00)	13.60 (13.00, 14.30)	13.50 (12.80, 14.30)	13.55 (13.00, 14.30)	0.495
Platelet (K/uL)	188.00 (145.00, 241.00)	197.00 (149.00, 232.00)	189.00 (146.00, 249.00)	185.50 (144.00, 236.00)	178.00 (144.50, 245.50)	0.741
Scr (mg/dL)	0.90 (0.80, 1.20)	0.90 (0.80, 1.10)	0.90 (0.70, 1.10)	0.90 (0.80, 1.10)	1.00 (0.80, 1.30)	0.001
BUN (mg/dL)	17.00 (13.00, 21.00)	16.00 (13.00, 21.00)	16.00 (12.00, 21.00)	16.00 (13.00, 21.00)	17.00 (14.00, 26.00)	0.012
Albumin (g/dL)	3.49 (3.00, 3.90)	3.50 (3.02, 3.91)	3.50 (3.09, 3.94)	3.48 (3.00, 3.89)	3.40 (2.90, 3.80)	0.148
FBG (mg/dL)	134.50 (115.00, 170.00)	118.00 (105.00, 136.00)	129.00 (113.00, 152.00)	137.00 (118.00, 171.00)	173.00 (138.50, 226.50)	<0.001
Potassium (mmol/L)	4.30 (3.90, 4.80)	4.20 (3.80, 4.70)	4.20 (3.90, 4.60)	4.30 (3.90, 4.90)	4.40 (3.90, 4.95)	0.040
Sodium (mmol/L)	137.00 (135.00, 139.00)	138.00 (135.00, 139.00)	137.00 (135.00, 139.00)	137.00 (135.00, 138.00)	136.00 (134.00, 138.00)	<0.001
TG (mg/dL)	131.02 (90.00, 196.00)	64.15 (48.00, 85.00)	119.00 (100.91, 137.00)	164.79 (130.63, 199.36)	230.00 (182.70, 308.15)	<0.001
TC (mg/dL)	162.26 (133.00, 191.00)	150.32 (120.34, 178.00)	163.00 (130.00, 189.25)	160.00 (137.00, 190.00)	177.00 (142.76, 205.52)	<0.001
LDL (mg/dL)	91.23 (67.00, 115.00)	88.00 (63.00, 116.19)	91.00 (70.73, 115.00)	91.00 (67.00, 112.46)	94.30 (66.00, 115.00)	0.811
HDL (mg/dL)	42.00 (35.00, 51.59)	46.00 (39.50, 57.00)	44.00 (36.00, 52.56)	40.51 (33.89, 50.00)	39.00 (31.00, 46.15)	<0.001
TyG index	9.15 (8.69, 9.58)	8.27 (8.02, 8.56)	8.95 (8.85, 9.04)	9.34 (9.26, 9.45)	9.89 (9.73, 10.18)	<0.001
AKI, *n* (%)	238 (30.13)	50 (25.38)	52 (26.13)	57 (29.38)	79 (39.50)	0.007

HR: heart rate; SBP: systolic blood pressure; DBP: diastolic blood pressure; RR: respiratory rate; SOFA: sequential organ failure assessment; CKD: chronic kidney disease; DM: diabetes mellitus; RRT: renal replacement therapy; RBC: red blood cell; WBC: white blood cell; RDW: red blood cell distribution width; SCr: serum creatinine; BUN: blood urea nitrogen; FBG: fasting blood glucose; TG: triglycerides; TC: total cholesterol; LDL: low-density lipoprotein; HDL: high-density lipoprotein; TyG: triglyceride glucose; AKI: acute kidney injury

The baseline signs between AKI and non-AKI individuals are shown in Table 2. AKI patients exhibited higher age, heart rate, respiratory rate, SOFA score, and lower blood pressure levels. In addition, AKI patients had a higher proportion of complications with heart failure, CKD, DM and sepsis, and were more likely to use vasoactive drugs and diuretics. In terms of laboratory parameters, AKI patients had higher WBC, RDW, Scr, BUN, FBG, and potassium levels, while their RBC, hemoglobin, platelets, albumin, sodium, TC, and LDL levels were lower than non-AKI patients.

**Table 2:** Baseline characteristics of the AKI and Non-AKI groups

Variables	Overall	AKI	Non-AKI	*P*-value
Participants	790	238	552	
Age (years)	67.65 (58.59, 76.99)	73.08 (62.61, 80.52)	65.77 (57.23, 73.64)	<0.001
Male, *n* (%)	567 (71.77)	167 (70.17)	400 (72.46)	0.511
BMI (kg/m^2^)	29.02 (25.72, 32.26)	29.00 (25.40, 32.35)	29.05 (25.81, 32.25)	0.721
Vital signs				
HR (beats/min)	79.68 (71.45, 87.04)	81.05 (74.83, 87.49)	78.84 (70.49, 86.81)	0.009
SBP (mmHg)	112.68 (105.68, 120.83)	109.28 (103.38, 117.00)	113.81 (106.69, 122.45)	<0.001
DBP (mmHg)	61.35 (55.00, 69.94)	58.01 (52.77, 64.93)	62.47 (56.85, 71.08)	<0.001
RR (beats/min)	18.08 (16.38, 20.11)	18.63 (16.72, 20.61)	17.89 (16.22, 19.84)	0.004
SOFA	3.00 (1.00, 6.00)	6.00 (3.00, 9.00)	2.00 (1.00, 4.00)	<0.001
Comorbidities, *n* (%)				
Heart failure	257 (32.53)	123 (51.68)	134 (24.28)	<0.001
CKD	119 (15.06)	60 (25.21)	59 (10.69)	<0.001
Hypertension	298 (37.72)	76 (31.93)	222 (40.22)	0.028
DM	125 (15.82)	54 (22.69)	71 (12.86)	0.001
Sepsis	287 (36.33)	142 (29.67)	145 (26.27)	<0.001
Malignancy	105 (13.29)	38 (15.97)	67 (12.14)	0.146
Interventions, *n* (%)				
RRT	40 (5.06)	40 (16.81)	0 (0.00)	<0.001
Diuretics	460 (58.23)	181 (70.05)	279 (50.54)	<0.001
Vasoactive drugs	169 (21.39)	103 (43.28)	66 (11.96)	<0.001
Laboratory tests				
RBC (m/uL)	3.76 (3.10, 4.35)	3.34 (2.79, 4.14)	3.91 (3.25, 4.46)	<0.001
WBC (K/uL)	11.50 (8.80, 15.00)	12.65 (9.70, 17.00)	11.10 (8.50, 14.40)	<0.001
Hemoglobin (g/dL)	11.80 (9.70, 13.50)	10.70 (8.70, 12.70)	12.20 (10.45, 13.70)	<0.001
RDW (%)	13.50 (12.90, 14.30)	13.80 (13.10, 14.90)	13.40 (12.90, 14.10)	<0.001
Platelet (K/uL)	188.00 (145.00, 241.00)	173.00 (136.00, 232.00)	195.50 (152.50, 242.50)	<0.001
Scr (mg/dL)	0.90 (0.80, 1.20)	1.10 (0.80, 1.50)	0.90 (0.70, 1.10)	<0.001
BUN (mg/dL)	17.00 (13.00, 21.00)	20.00 (14.00, 27.00)	16.00 (13.00, 20.00)	<0.001
Albumin (g/dL)	3.49 (3.00, 3.90)	3.40 (2.90, 3.80)	3.50 (3.04, 3.91)	0.004
FBG (mg/dL)	134.50 (115.00, 170.00)	149.00 (120.00, 198.00)	131.00 (111.50, 159.00)	<0.001
Potassium (mmol/L)	4.30 (3.90, 4.80)	4.50 (3.90, 5.00)	4.20 (3.90, 4.65)	<0.001
Sodium (mmol/L)	137.00 (135.00, 139.00)	136.00 (134.00, 138.00)	137.00 (135.00, 139.00)	<0.001
TG (mg/dL)	131.02 (90.00, 196.00)	134.50 (92.00, 197.27)	130.15 (89.50, 193.69)	0.651
TC (mg/dL)	162.26 (133.00, 191.00)	157.13 (124.00, 187.00)	165.00 (134.40, 193.66)	0.011
LDL (mg/dL)	91.23 (67.00, 115.00)	86.32 (60.29, 108.80)	93.00 (69.00,116.10)	0.005
HDL (mg/dL)	42.00 (35.00, 51.59)	43.28 (33.06, 51.59)	42.00 (35.00, 51.71)	0.510
TyG index	9.15 (8.69, 9.58)	9.26 (8.78, 9.77)	9.11 (8.66, 9.51)	0.002

HR: heart rate; SBP: systolic blood pressure; DBP: diastolic blood pressure; RR: respiratory rate; SOFA: sequential organ failure assessment; CKD: chronic kidney disease; DM: diabetes mellitus; RRT: renal replacement therapy; RBC: red blood cell; WBC: white blood cell; RDW: red blood cell distribution width; SCr: serum creatinine; BUN: blood urea nitrogen; FBG: fasting blood glucose; TG: triglycerides; TC: total cholesterol; LDL: low-density lipoprotein; HDL: high-density lipoprotein; TyG: triglyceride glucose; AKI: acute kidney injury

### Primary endpoints

**Fig. 2:**
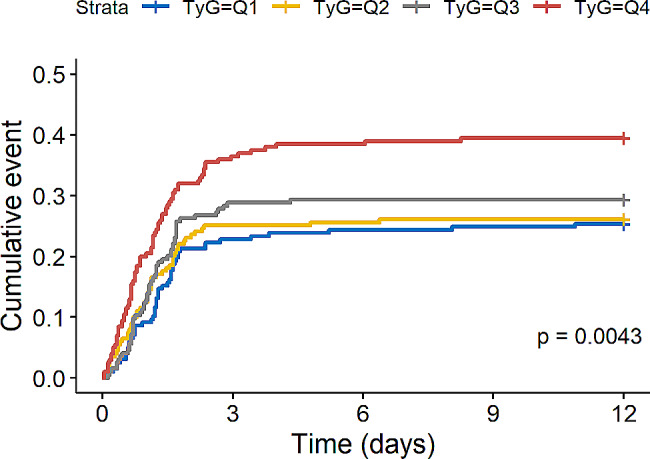
Cumulative event incidence curve for AKI incidence. (TyG index quartile Q1: 5.17–8.69; Q2: 8.69–9.15; Q3: 9.15–9.58; Q4: 9.58–11.44)

Of the 790 patients, 238 (30.13%) developed AKI. Figure 2 showed differences in cumulative AKI incidence among the four groups. The incidence of AKI also increased progressively with increasing quartiles of TyG index (log-rank *P* = 0.0043).

As shown in Table 3, multivariate Cox regression models confirmed the independent impact of TyG index on the incidence of AKI in patients with coronary revascularization. Cox proportional hazards analysis showed that TyG index was a significant risk factor for AKI in the unadjusted model (HR, 1.29 [95% CI 1.08–1.54], *P* = 0.005), partially adjusted model (HR, 1.40 [95% CI 1.16–1.68], *P* < 0.001), and fully adjusted model (HR, 1.42 [95% CI 1.06–1.92], *P* = 0.018) when TyG index was treated as a continuous variable. In addition, when TyG index was considered as a nominal variable, patients in the higher quartile (Q4) of TyG index were significantly associated with AKI risk: unadjusted model (HR, 1.75 [95% CI 1.23–2.49], *P* = 0.002), partially adjusted model (HR, 2.00 [95% CI 1.39–2.88], *P* < 0.001), and fully adjusted model (HR, 1.89 [95% CI 1.12–3.17], *P* = 0.017). Compared with subjects in the lowest quartile, the risk of AKI showed an increasing trend with increasing TyG index.

**Table 3:** Cox proportional hazard ratios for AKI incidence

Categories	Model 1 HR (95% CI)	*P*-value	Model 2 HR (95% CI)	*P*-value	Model 3 HR (95% CI)	*P*-value
AKI incidence						
TyG *	1.29 (1.08–1.54)	0.005	1.40 (1.16–1.68)	<0.001	1.42 (1.06–1.92)	0.018
TyG (category)						
Q1 (5.17–8.69)	Ref.		Ref.		Ref.	
Q2 (8.69–9.15)	1.06 (0.72–1.56)	0.765	1.11 (0.75–1.64)	0.592	1.36(0.90–2.06)	0.143
Q3 (9.15–9.58)	1.20 (0.82–1.75)	0.346	1.14 (0.90–1.95)	0.147	1.51 (0.96–2.36)	0.071
Q4 (9.58–11.44)	1.75 (1.23–2.49)	0.002	2.00 (1.39–2.88)	<0.001	1.89 (1.12–3.17)	0.017
*P* for trend		0.001		<0.001		0.019

*Continuous variable per 1 unit

Model 1 was unadjusted

Model 2 was adjusted for sex, age, and BMI

Model 3 was adjusted for sex, age, BMI, albumin, Scr, BUN, WBC, RBC, platelet, hemoglobin, RDW, LDL, HDL, TG, vasoactive drugs, diuretics, hypertension, heart failure, DM, CKD, and RRT

**Fig. 3:**
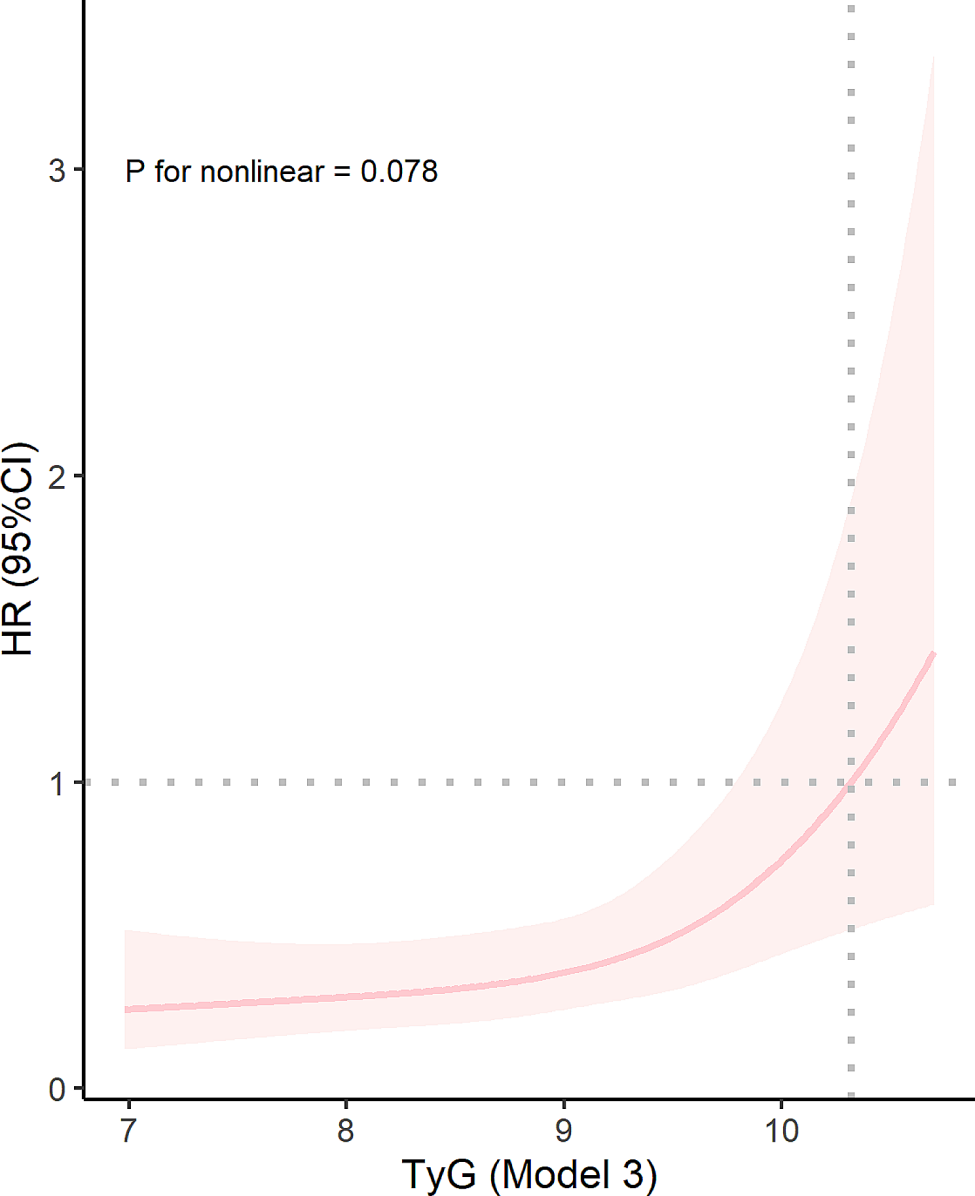
Restricted cubic spline curve of TyG index with AKI incidence

Besides, the dose-response relationship between TyG index and AKI risk was explored using RCS regression models. The RCS regression curve (Fig. 3) showed a linear association between TyG index and AKI after adjusting for confounders (*P* for non-linearity = 0.078).

**Fig. 4:**
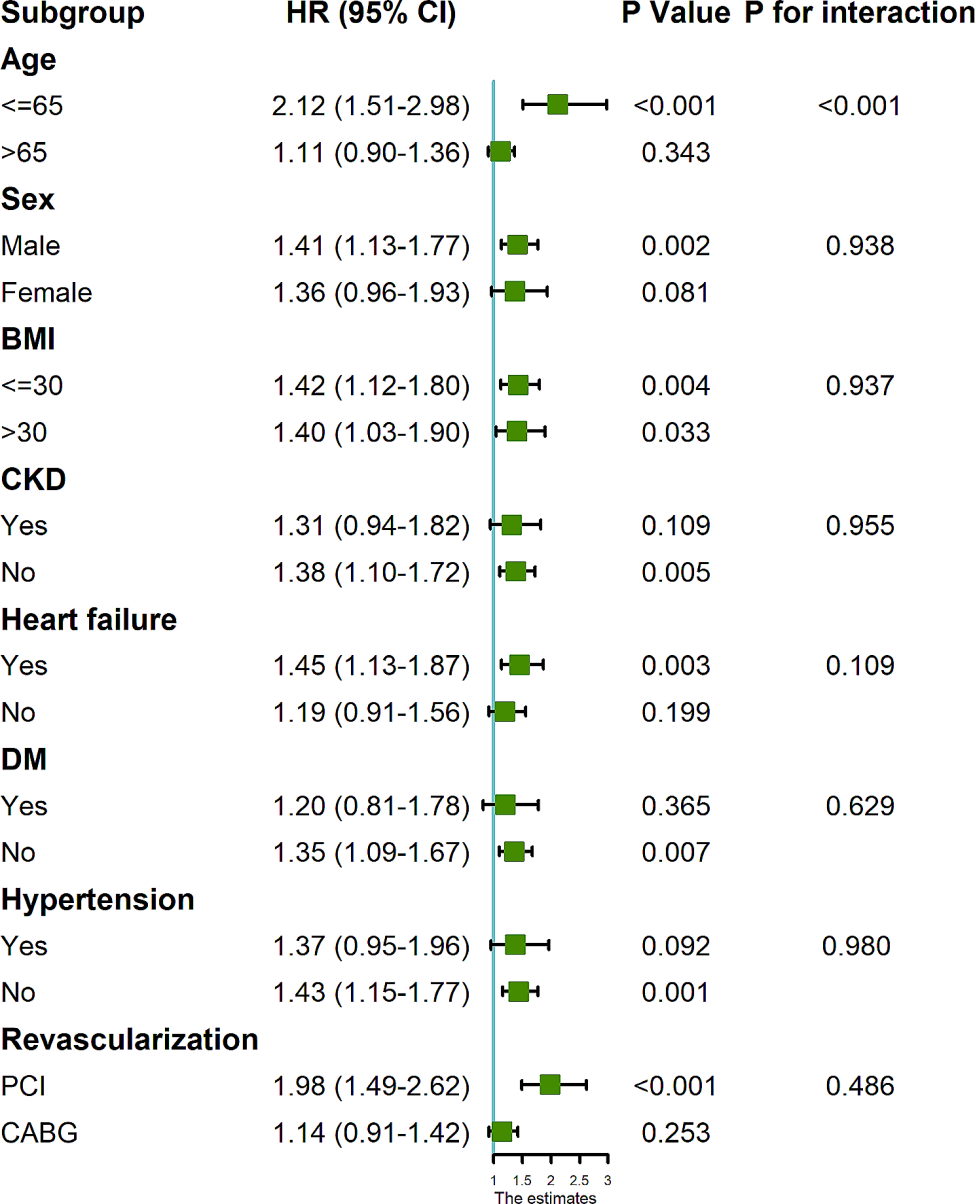
Forest plots for the primary endpoint in different subgroups

Further stratification and interaction analyses of the relationship between TyG index (continuous variable per 1 unit) and AKI according to potential confounders such as age, sex, BMI, CKD, heart failure, DM, and hypertension (Fig. 4) showed that TyG index was significantly associated with the risk of AKI in the subgroup of patients with coronary revascularization, which included patients younger than 65 years (HR, 2.12 [95% CI 1.51–2.98], *P* < 0.001), male (HR, 1.41 [95% CI 1.13–1.77], *P* = 0.002), patients with BMI less than 30 (HR, 1.42 [95% CI 1.12–1.80], *P* = 0.004), patients with BMI greater than 30 (HR, 1.40 [95% CI 1.03–1.90], *P* = 0.033), patients without CKD (HR, 1.38 [95% CI 1.10–1.72], *P* = 0.005), patients with heart failure (HR, 1.45 [95% CI 1.13–1.87], *P* = 0.003), patients without DM (HR, 1.35 [95% CI 1.09–1.67], *P* = 0.007), patients without hypertension (HR, 1.43 [95% CI 1.15–1.77], *P* = 0.001), and patients with PCI (HR, 1.98 [95% CI 1.49–2.62], *P* < 0.001). No significant interaction effect was observed between TyG index and AKI risk, except for age (*P* for interaction < 0.001). The association between TyG index and AKI seems to be more prominent in patients younger than 65 years.

### Secondary endpoints

**Fig. 5:**
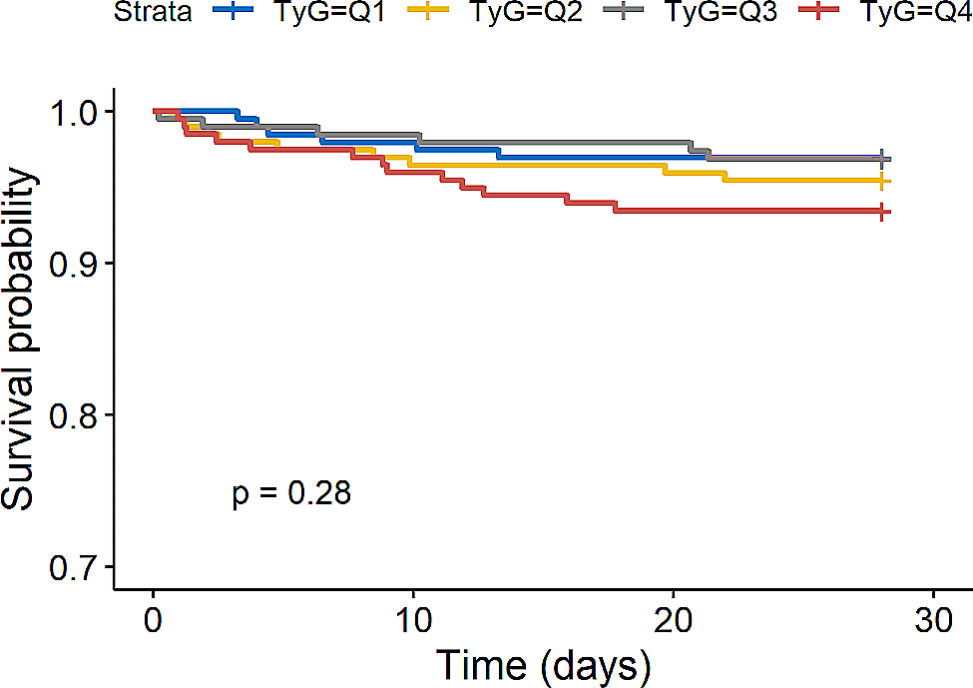
Kaplan-Meier survival analysis curve for 28-day mortality in the overall study population. (TyG index quartile Q1: 5.17–8.69; Q2: 8.69–9.15; Q3: 9.15–9.58; Q4: 9.58–11.44)

**Fig. 6:**
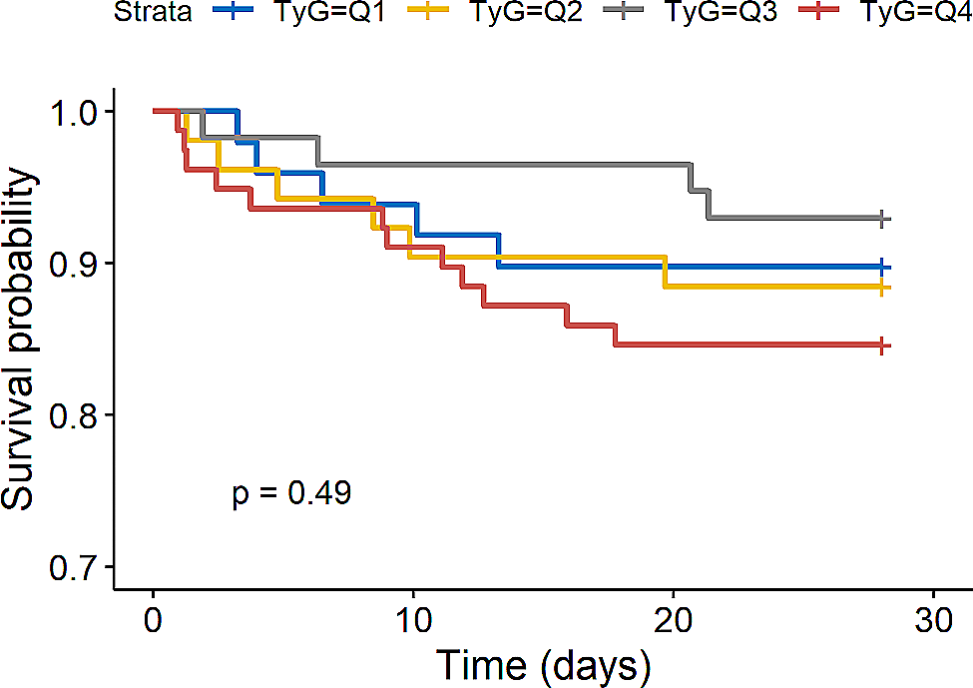
Kaplan-Meier survival analysis curve for 28-day mortality in AKI population. (TyG index quartile Q1: 5.17–8.69; Q2: 8.69–9.15; Q3: 9.15–9.58; Q4: 9.58–11.44)

**Fig. 7:**
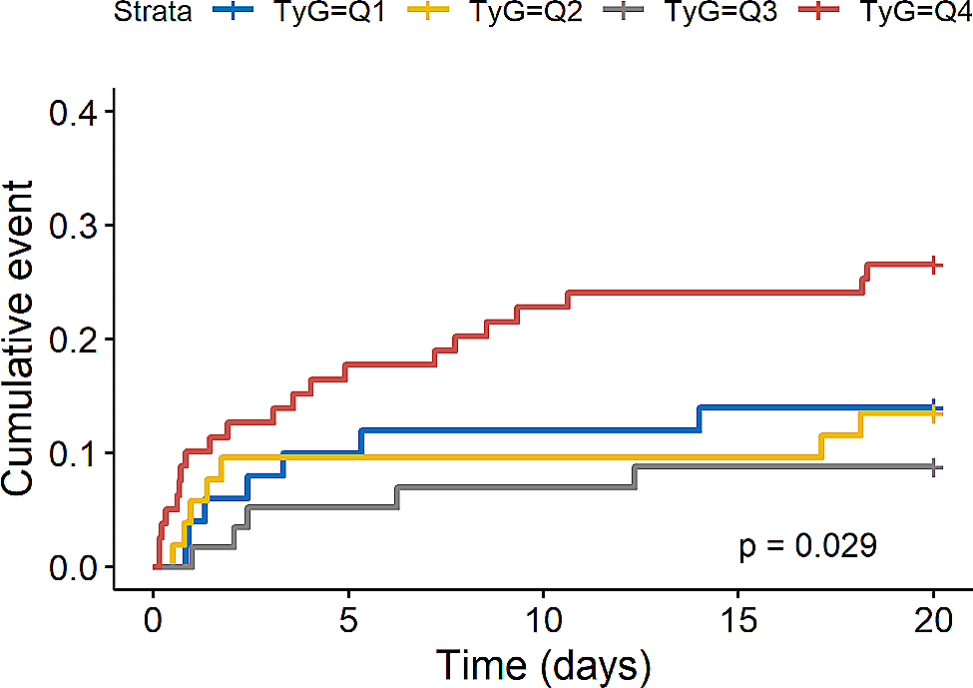
Cumulative event incidence curves for the RRT use in AKI population. (TyG index quartile Q1: 5.17–8.69; Q2: 8.69–9.15; Q3: 9.15–9.58; Q4: 9.58–11.44)

We further evaluated the impact of TyG index on secondary endpoints such as 28-day mortality in the overall study population and AKI population and the rate of RRT application in AKI population. Kaplan-Meier survival analysis showed that TyG index was not significantly associated with 28-day mortality in the overall study population (*P* = 0.28, Fig. 5) and AKI population (*P* = 0.49, Fig. 6). Interestingly, we found that AKI patients in the higher TyG index quartile (Q4) had a significantly increased risk of applying RRT (*P* = 0.029, Fig. 7).

## Discussion

CAD seriously endangers public health and life safety, and CABG and PCI are currently recognized as superior evidence-based strategies for CAD. However, AKI is a common complication of CABG and PCI. Early identification of AKI risk remains an extremely challenging hot topic in the field of coronary revascularization. Our study found that elevated TyG index was associated with an increased risk of AKI in patients with CABG and/or PCI surgery, and high levels of TyG index were an independent risk factor for AKI. This association held even after accounting for potential confounding factors. The RCS model suggested that the risk of AKI in patients with coronary revascularization was linearly correlated with TyG index. In addition, our study also found that patients who developed AKI after coronary revascularization with a high TyG index faced a higher risk of RRT. To our knowledge, this is the first study to investigate the relationship between different TyG indices and AKI in patients with coronary revascularization. In conclusion, this study provides a novel, simple, and efficient biomarker for risk stratification of AKI in patients with coronary revascularization, and the TyG index may be a promising decision-making tool for clinicians.

Insulin metabolic signaling regulates cardiovascular remodeling and blood pressure by affecting the sympathetic nervous system, renin-angiotensin-aldosterone system (RAAS), and intrarenal regulatory mechanisms. However, IR is responsible for a range of metabolic and hemodynamic abnormalities. The presence of IR is central to cardio-renal metabolic syndrome, and is increasingly recognized as an independent risk factor for cardiovascular disease and kidney disease, independent of DM [20, 21]. TyG index is a substitute index recently used to assess IR, and it has been shown to be superior to homeostasis model assessment of IR in patients with or without DM [22, 23]. Recent studies have found that TyG index is closely associated with the incidence, severity, adverse events, and mortality of multiple cardio-renal diseases.

In cardiovascular disease, a meta-analysis including 41 studies showed that patients with higher TyG index levels had a higher risk of CAD, more severe CAD, and worse cardiovascular prognosis compared with patients with lower TyG index levels [24]. Huang et al. found that the higher the TyG index of subjects without previous heart failure and coronary heart disease, the higher risk of developing heart failure, and the higher risk of poor left ventricular remodeling and dysfunction in a community-based cohort [3]. Furthermore, Liu et al. reported that TyG index > 9.20 was an independent risk factor for atrial fibrillation in the general population without known cardiovascular disease [25]. It is worth noting that TyG index also has clinical predictive value for coronary revascularization. Ma et al. found that TyG index was independently associated with adverse cardiovascular outcomes such as mortality, non-fatal myocardial infarction, and non-fatal stroke in T2DM patients with acute coronary syndrome undergoing PCI [26]. Similarly, Wu et al. revealed a significant association between TyG index and adverse cardiovascular outcomes in a cohort of more than 1000 patients with CABG [27].

In terms of kidney disease, Yang et al. found a significant linear relationship between TyG index levels and the risk of AKI in patients with critical heart failure, and an increase in TyG index predicted a higher RRT usage rate in patients with heart failure and AKI [18]. Similarly, Jin et al. found a higher TyG index in critically ill patients was significantly correlated with increased susceptibility to AKI through a large cross-sectional study [28]. In addition, the relationship between TyG index and chronic renal deterioration has also been concerned. Gao et al. revealed that TyG index of T2DM and CKD was positively associated with the risk of end-stage renal disease [29]. Ou et al. and Low et al. found that TyG index was closely related to proteinuria and kidney disease progression in T2DM [30, 31]. The evidence collectively highlights the TyG index as a reliable and independent predictor of adverse renal outcomes, which may contribute to risk stratification of AKI in patients with coronary revascularization.

Current evidence has suggested that the TyG index may be a valid predictor of AKI risk in patients with heart failure, critically ill patients, and worsening renal function in DM and CKD. However, the predictive ability of the TyG index for AKI risk in patients with coronary revascularization remains unclear. Our study revealed that TyG index was significantly and independently associated with the incidence and severity of AKI in patients with coronary revascularization. Recent studies have found that IR plays a crucial role in this cardio-renal syndrome pathology, and the possible mechanisms include the following aspects. First, IR is associated with increased glomerular hydrostatic pressure, resulting in impaired glomerular filtration, increased permeability, and hyperfiltration [32]. In addition, IR causes the loss of podocyte foot process cytoskeletal structure, resulting in abnormal albuminuria excretion and glomerular hyperfiltration [33]. Second, IR-induced insulin compensatory increase has adverse consequences on cardio-renal tissue. Excess insulin contributes to mesangial proliferation and tubulointerstitial fibrosis, thereby exacerbating renal tissue damage [34]. Third, IR can increase the levels of angiotensin II and aldosterone, which may be associated with an imbalance in the sympathetic nervous system and RAAS. Angiotensin II causes increased blood pressure and renal vasoconstriction, leading to decreased perfusion of renal tissue [35]. Meanwhile, both angiotensin II and aldosterone promote the activation of multiple redox-sensitive kinases, generating podocyte injury and renal tissue remodeling [36, 37]. Fourth, IR leads to a decrease in PI3K/Akt, which results in a decrease in nitric oxide (NO) production in glomerular endothelial cells, contributing to their associated tubuloglomerular feedback, hyperfiltration and sodium retention, ultimately affecting cardiac afterload, renal endothelial function and renal hemodynamics [38, 39]. Moreover, reduced NO facilitates the recruitment of leukocytes and monocytes, platelet aggregation and inflammatory responses [40]. Fifth, IR abnormally activates certain signaling pathways, such as inflammation, oxidative stress, endoplasmic reticulum stress, and abnormal activation of these signaling pathways together accelerates glomerular and interstitial damage and renal insufficiency [41]. In conclusion, the TyG index, a reliable and effective marker for evaluating IR, is conducive to optimizing the risk stratification of AKI in patients with coronary revascularization, thereby guiding early clinical intervention.

However, some limitations remain in our study. First, our study is a retrospective, single-center cohort study with a relatively small sample size, which needs to be further verified by multi-center, large-sample prospective studies. Second, we investigated the relationship between initial TyG index after admission and AKI risk in patients with coronary revascularization, and further evaluation of the predictive value of dynamic TyG index for AKI risk is needed in the future. In addition, the prognostic mechanisms of TyG index for AKI risk stratification need to be further investigated. Third, baseline levels of FPG and TG may be influenced by the use of lipid-lowering drugs and antidiabetic drugs prior to admission. However, the use of lipid-lowering drugs and hypoglycemic drugs was not considered in this study, as the data on drugs used before admission were relatively limited, which may cause potential bias in the study results.

## Conclusion

High TyG index is an independent risk factor for AKI and adverse renal outcomes in patients with coronary revascularization. And TyG index is a simple and efficient biomarker for AKI risk stratification.

## Data Availability

Data will be made available from the corresponding author on reasonable request.

## Abbreviations

TyG: Triglyceride glucose
AKI: Acute kidney injury
MIMIC-IV: Medical information mart for intensive care IV
RRT: Renal replacement therapy
RCS: Restricted cubic splines
CAD: Coronary artery disease
PCI: Percutaneous coronary intervention
CABG: Coronary artery bypass grafting
IR: Insulin resistance
FBG: Fasting blood glucose
TG: Triglycerides
T2DM: Type 2 diabetes
Scr: Serum creatinine
BMI: body mass index
SOFA: Sequential organ failure assessment
WBC: White blood cell
RBC: Red blood cell
RDW: Red blood cell distribution width
BUN: Blood urea nitrogen
TC: Total cholesterol
LDL: Low-density lipoprotein
HDL: High-density lipoprotein
CKD: Chronic kidney disease
DM: Diabetes mellitus
HR: Hazard ratio
CI: Confidence interval
RAAS: Renin-angiotensin-aldosterone system
NO: Nitric oxide
